# Interlocking plate compared with conventional pin fixation for undisplaced femoral neck fractures: a randomized controlled trial with radiostereometric analysis

**DOI:** 10.1302/2633-1462.79.BJO-2026-0008.R1

**Published:** 2026-09-04

**Authors:** Magnus Høgevold, Michaela Manalili Hansen, Jan Egil Brattgjerd, Stephan Maximillian Röhrl

**Affiliations:** 1 Division of Orthopedic Surgery, Diakonhjemmet Hospital, Oslo, Norway; 2 Institute of Clinical Medicine, Faculty of Medicine, University of Oslo, Oslo, Norway; 3 Department of Orthopedic Surgery and Traumatology, Odense University Hospital, Odense, Denmark; 4 Department of Clinical Medicine, University of Southern Denmark, Odense, Denmark; 5 Division of Orthopedic Surgery, Oslo University Hospital, Oslo, Norway; 6 Faculty of Health Sciences, Oslo Metropolitan University, Oslo, Norway

**Keywords:** Femoral neck fracture, Internal fixation, Radiostereometric analysis (RSA), Hansson pinloc system, Hansson pins, femoral neck fractures, pin fixation, randomized controlled trial, trochanteric fractures, clinical outcomes, plate fixation, Avascular necrosis, reoperations, Numeric Rating Scale

## Abstract

**Aims:**

In undisplaced femoral neck fractures, fixation failure remains a concern. In vitro studies suggest that interlocking plates increase torsional stability. However, whether this leads to clinically relevant improvement in fixation stability in vivo remains unclear. Accordingly, we tested whether interlocking plate fixation provides increased in vivo fixation stability over 52 weeks in undisplaced femoral neck fractures.

**Methods:**

Having been recruited from the Emergency Departments of two hospitals between May 2016 and January 2019, patients were randomized to Hansson pin fixation (n = 12) or Hansson Pinloc plate fixation (n = 13). Postoperative fracture motion was quantified using radiostereometric analysis (RSA) at baseline and at four, 12, 26, and 52 weeks. Clinical outcomes (Harris Hip Score, EuroQol five-dimension (EQ-5D) questionnaire, Timed Up and Go test, and pain and satisfaction using the Numeric Rating Scale) were assessed at the same timepoints. Data were analyzed using linear mixed model to account for missing RSA points.

**Results:**

A total of 17 patients completed the 52-week follow-up. The Pinloc group showed a smaller mean total rotation than the pin group, with a mean difference of −7.77° (95% CI −13.27° to −2.28°; p = 0.006) at 52 weeks. The groups diverged significantly in torsional stability at 12 weeks, with a larger between-group difference during follow-up. No between-group differences were observed in total translation or clinical outcomes. Five patients required reoperation: three in the Pinloc group and two in the pin group. Avascular necrosis occurred in three patients. Sub-trochanteric fractures occurred in two patients, both in the Pinloc group.

**Conclusion:**

Interlocking plate fixation resulted in increased torsional stability compared with pin fixation during healing of undisplaced femoral neck fractures, consistent with previous in vitro studies. The clinical implications of this finding remain uncertain.

Cite this article: *Bone Jt Open* 2026;7(9):1154–1162.

## Introduction

Internal fixation with screws or pins is the most common treatment of undisplaced femoral neck fractures in older patients.^1,2^ Despite decades of use, no conventional implant has demonstrated clear superiority,^3^ consistent with similar biomechanical performance in radiostereometric analysis (RSA) studies.^4,5^

Although most of these fractures are generally considered stable, reoperation rates are not negligible (7% to 14%), and are often associated with suboptimal clinical outcomes.^1,6,7^ Consequently, various locking plate designs have been introduced to increase stability, although their biomechanical necessity in stable fractures remains uncertain. While clinical outcomes are commonly used, in vivo biomechanical evaluation may better explain clinical performance.

**Fig. 1:**
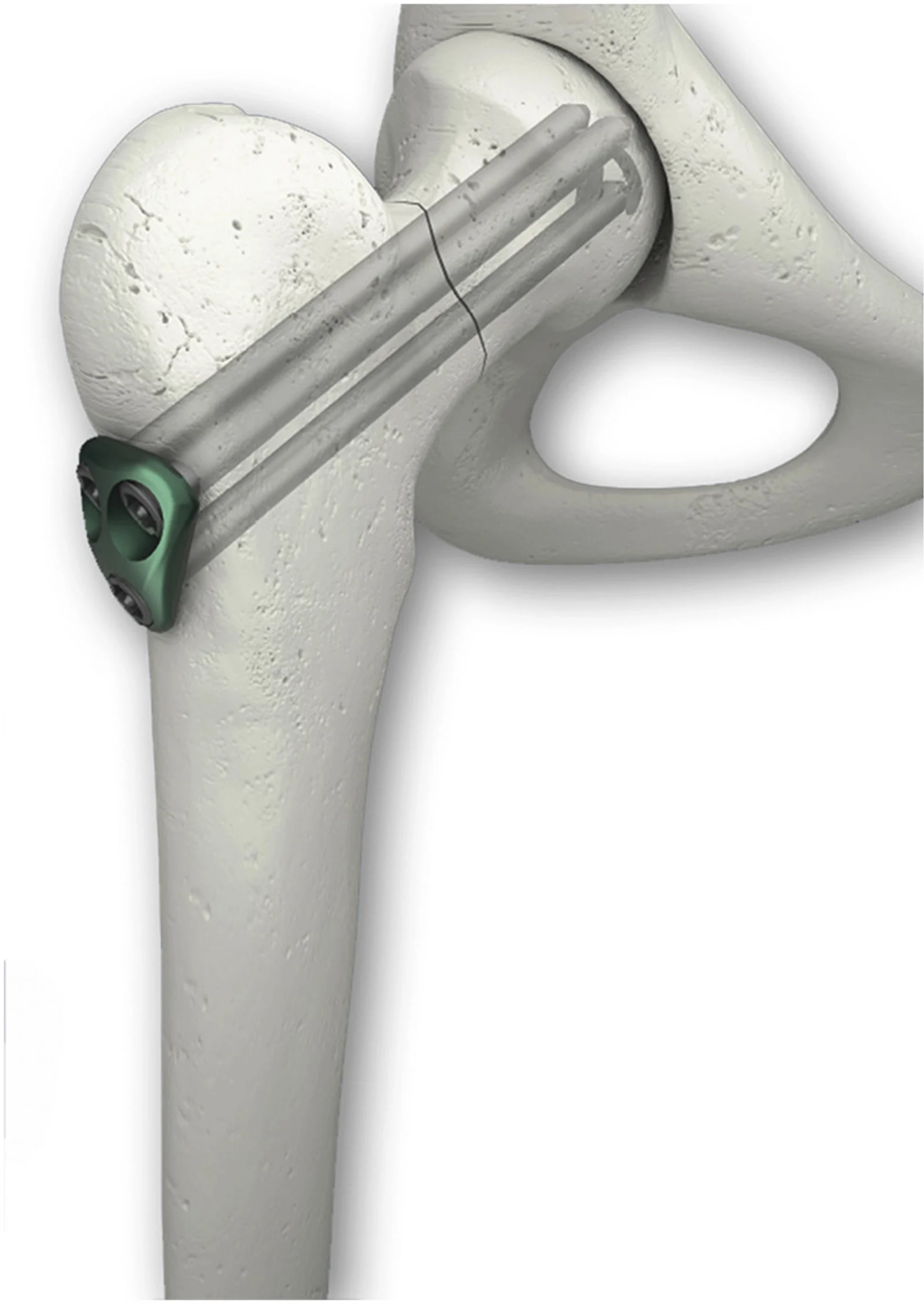
Hansson Pinloc System (illustration from Swemac Innovation, Sweden).

While plate designs have shown promising clinical results,^8^ mechanical failure modes have also been reported.^9,10^ The Hansson Pinloc system (Swemac Innovations, Sweden) (Figure 1) combines a small locking plate with the principles of Hansson pins, originally used in pairs (Swemac Innovations). Preclinical studies suggest primarily improved torsional stability ex vivo*,*^11,12^ and previous RSA data from displaced femoral neck fractures have linked increased fracture motion to higher risk of nonunion.^4^ Consequently, whether interlocking pins can increase fixation stability and prevent fixation failure remains unknown,^13^ as their biomechanical performance has not been characterized using RSA.

The aim of this study was therefore to compare postoperative fracture motion over 52 weeks between the Hansson Pinloc system and traditional Hansson pins, using RSA, in patients with undisplaced femoral neck fractures.

## Methods

### Trial design and ethics

This two-centre, randomized superiority trial at Oslo University Hospital, Ullevål and Diakonhjemmet Hospital, Norway, (ClinicalTrials.gov: NCT02699619) was approved by the Regional Ethics Committee South-East Norway B (2015/191) and reported following the CONSORT statement. All patients received written and verbal information and provided written informed consent. No external funding was received. De-identified data and statistical code are available on request.

### Participants

Patients were recruited in the Emergency Departments of both hospitals between May 2016 and January 2019.

Inclusion criteria were age ≥ 60 years, Garden type I to II femoral neck fracture, ability to walk independently, provide informed consent, and fitness for surgery. Exclusion criteria were a previous fracture of the same hip, unwillingness or inability to attend follow-up, or severe comorbidity with limited life expectancy. All included patients had a posterior tilt < 20° on lateral radiographs,^14^ and were American Society of Anesthesiologists (ASA) grade II or III.^15^

No formal screening log was kept, and all patients who were invited to participate consented and were enrolled in the study.

Baseline characteristics were similar between groups (Table I).

**Table I.** Patient demographic details.

Variable	Pin group	Pinloc group	p-value
Total, n	12	13	
Female, n (%)	9 (75)	8 (62)	0.673*
Median age, yrs (IQR)	79.5 (73 to 83)	76 (72 to 86)	0.698†
**ASA grade, n (%)**			0.238*
II	4 (33)	8 (62)	
III	8 (67)	5 (39)	
Side: Left, n	6	4	0.428*
Median T-score hip (IQR)	−1.7 (−3.3 to −1.3)	−2.4 (−3.4 to −1.5)	0.424†

* Fisher’s exact test.

† Mann-Whitney U test (exact p-value).

ASA, American Society of Anesthesiologists.

### Randomization and allocation concealment

Patients were randomized 1:1 to Hansson Pinloc plate fixation (Pinloc group) or Hansson pin fixation (pin group) using computer-generated block randomization with a block size of four and no stratification.

To ensure concealment, sequentially numbered, opaque envelopes were prepared and stored by the research coordinator (see Acknowledgements) and opened by the surgeon in the operating theatre before surgery.

### Interventions

Procedures were performed at both hospitals. One study surgeon (JEB, MH) was assigned to each hospital.

Before study start, surgeons completed a structured training module provided by the implant manufacturer (Swemac Innovations), which included a simulated operation with implant placement, fluoroscopy, and implant selection, regardless of prior experience with the implants. Certification was required before independent operating in the trial.

The fractures were fixated in situ on a traction table under fluoroscopic guidance. Fracture position was verified before fixation to ensure no secondary displacement had occurred; no patients were excluded intraoperatively for this reason. All procedures followed the manufacturer’s operative manuals, with an open lateral approach. In the Pinloc group, fixation was performed with plate size determined intraoperatively to suit the patient’s anatomy; the plate had a fixed 125° angle. In the pin group, pin distance was set under fluoroscopy. Perioperative care was identical for both groups.

### Radiostereometric analysis

Before implant insertion, eight to ten tantalum markers (1 mm) were placed in the femoral head and trochanteric region using a dedicated insertion device (UmRSA, RSA Biomedical, Sweden). Imaging was performed supine using a uniplanar calibration cage (no. 43, UmRSA, RSA Biomedical). At Oslo University Hospital, ceiling-mounted X-ray tubes were used; at Diakonhjemmet Hospital, both ceiling-mounted and portable tubes were used. The imaging setup was standardized across sites.

Analyses were carried out in RSAcore 4.0 (RSAcore, Leiden University Medical Center, Netherlands) using marker-based RSA with all-markers method along the global coordinate system. The trochanteric region was defined as the fixed segment and the femoral head as the moving segment. Images with a condition number (CN) > 150 or mean error (ME) of rigid body fitting > 0.35 mm were excluded.^16^ Double examinations were performed in all patients at least once to assess measurement error. In RSA, precision describes how closely repeated measurements agree under the same conditions. Precision was assessed using double examinations taken on the same day, with repositioning of the patient and the radiological setup between examinations. Assuming no migration between the examinations, precision was calculated as the SD of the differences between paired measurements, in accordance with current RSA guidelines.^16^ If RSA quality criteria were not met for a given examination, that timepoint was excluded.

RSA was chosen as the primary measurement method for its high precision in detecting small implant and fracture migrations.^17^

### Postoperative care and follow-up

Patients were mobilized by the hospital’s physiotherapy service and allowed weightbearing as tolerated. Baseline RSA was performed post-weightbearing, before hospital discharge (mean 3.3 days (SD 1.9)). RSA and clinical follow-ups were scheduled at four, 12, 26, and 52 weeks. In addition, seven patients had RSA images from before mobilization to assess potential initial settling; these were limited due to clinical priority of early mobilization.

Bone mineral density (BMD) was measured postoperatively using dual-energy X-ray absorptiometry (DXA) in an outpatient setting (Lunar Prodigy (GE Healthcare, USA) at Oslo University Hospital, and DiscoveryA (Hologic, USA) at Diakonhjemmet Hospital).

At each follow-up, patient-reported outcome measures (PROMs) were collected: Harris Hip Score (HHS),^18^ EuroQol five-dimension (EQ-5D) questionnaire,^19^ Timed Up and Go (TUG) test, pain (Numeric Rating Scale, NRS), and satisfaction (NRS). Complications were recorded continuously.

### Outcomes

The primary outcome was fracture migration (rotation and translation) at 52 weeks relative to baseline, measured by RSA.

Secondary outcomes were fracture motion during healing measured by RSA, time to healing, time of surgery reoperations of any reason, mortality, and PROMs (HHS, EQ-5D, TUG, NRS pain and satisfaction). Fracture healing was defined mechanically as the stabilization of migration below the RSA detection limit between two consecutive assessments. This was correlated with a visual inspection of the RSA radiographs, defining union as obliteration of the fracture line with pain-free weightbearing.

### Sample size

The sample size was based on RSA precision. A between-group difference of 2.0 mm was considered clinically relevant and detectable with 80% power at a two-sided α level of 0.05, assuming a measurement precision of 0.1 mm to 0.2 mm for translation and 0.2° to 0.6° for rotation.^20,21^ With an expected precision of 0.3 mm, eight patients per group were required.^22^ To allow for image quality issues and loss to follow-up, the number was increased to 15 per group.

### Statistical analysis

Baseline demographic details, clinical characteristics (sex, age, ASA grade, DXA) and operating time are present as frequencies and percentages for categorical variables, or median and IQR for continuous variables. Between-group comparisons were performed using Fisher’s exact test or Mann-Whitney U test as appropriate. The data were analyzed with Stata SE v. 19 (StataCorp, USA). Total rotation was calculated using Euler’s formula.

RSA migrations and PROMs were analyzed using linear mixed models (LMM) with time, group, and their interactions as fixed effects, and a subject-specific random intercept.^16^ Association between BMD and fracture migration was evaluated with a LMM with total translation and total rotation as dependent variables and time and BMD as fixed effects. Missing data at follow-up points were implicitly handled by the LMM, allowing all available data to contribute to the analysis without excluding participants under the missing-at-random assumption. The model used restricted maximum likelihood (REML) with Kenward-Roger degrees of freedom to provide accurate estimates, in spite of our small sample size, at the 52-week endpoint.

Analyses from LMM are presented as least squares means with 95% CIs; p-values < 0.05 were interpreted as statistically significant. Analyses were conducted according to the intention-to-treat principle.

## Results

### Participant flow and baseline

**Fig. 2:**
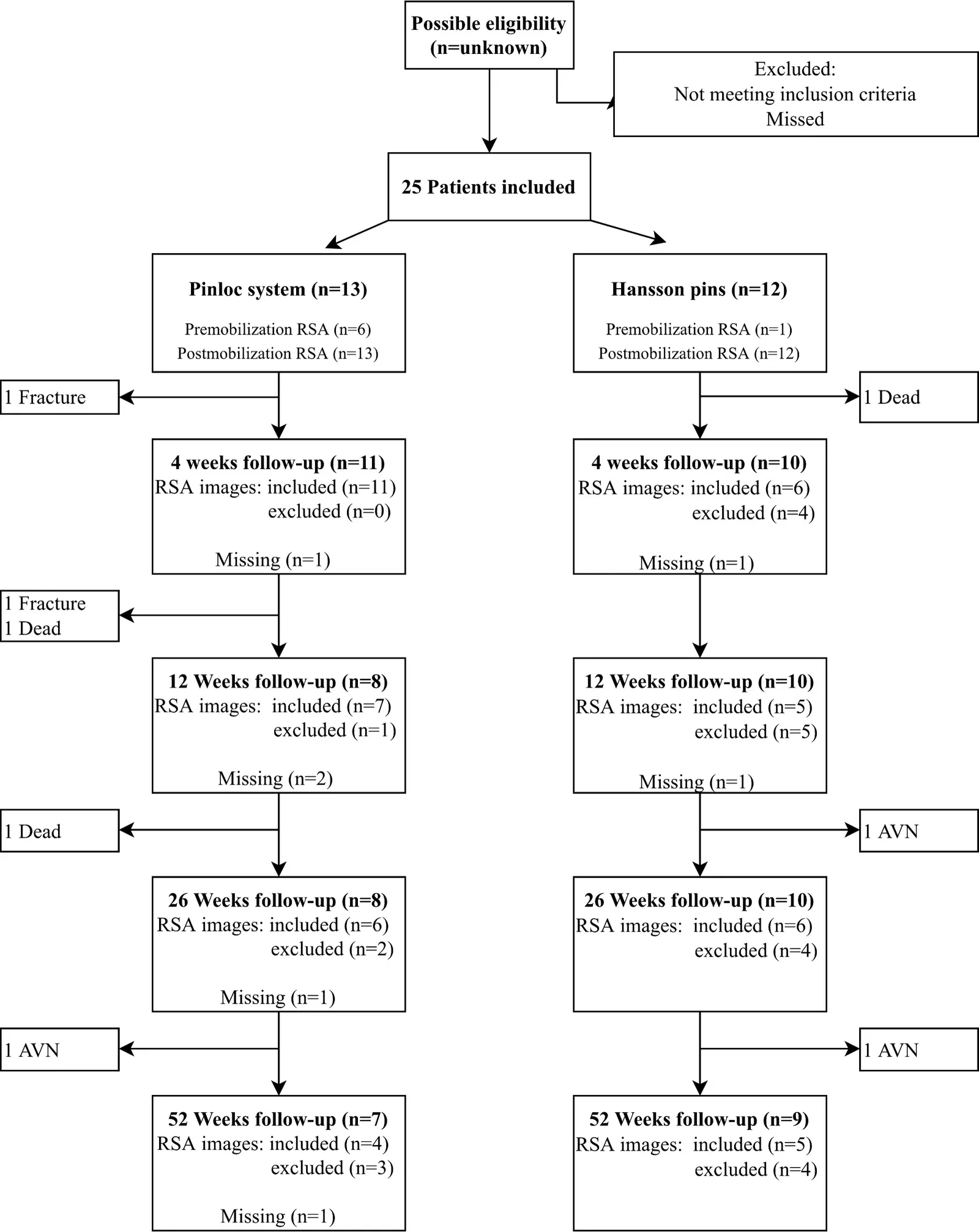
Flowchart. Radiostereometric analysis (RSA) image excluded if condition number > 150 or mean error of rigid body fitting > 0.35. AVN, avascular necrosis.

A total of 25 patients were included, 13 in the Pinloc group and 12 in the pin group (Figure 2). After the minimum sample size required for the primary outcome had been achieved, recruitment was discontinued because of a low recruitment rate.

### Radiostereometric analysis

Precision was based on 37 double examinations and ranged from 0.27 mm to 0.54 mm for translations and 0.44° to 1.15° for rotations (Table II).

**Table II.** Precision in translation and rotation based on 37 double examinations.

Fracture translation, mm	Mean	SD	Detection limit* (SD × 2.03)
X-axis (medial/lateral)	0.00	0.13	0.27
Y-axis (proximal/distal)	0.00	0.18	0.36
Z-axis (anterior/posterior)	0.03	0.27	0.54
Total translation	0.24	0.25	0.50
**Fracture rotation, °**			
X-axis (anterior/posterior)	-0.03	0.41	0.84
Y-axis (ante-/retroversion)	-0.16	0.57	1.15
Z-axis (varus/valgus)	-0.05	0.22	0.44
Total rotation	0.63	0.41	0.83

* Assuming no migration between the examinations, detection limit was calculated by multiplying the SD with the critical value (2.03; *α* = 0.05) obtained from the t-distrubution, corresponding to the degree of freedom (n – 1).

**Fig. 3:**
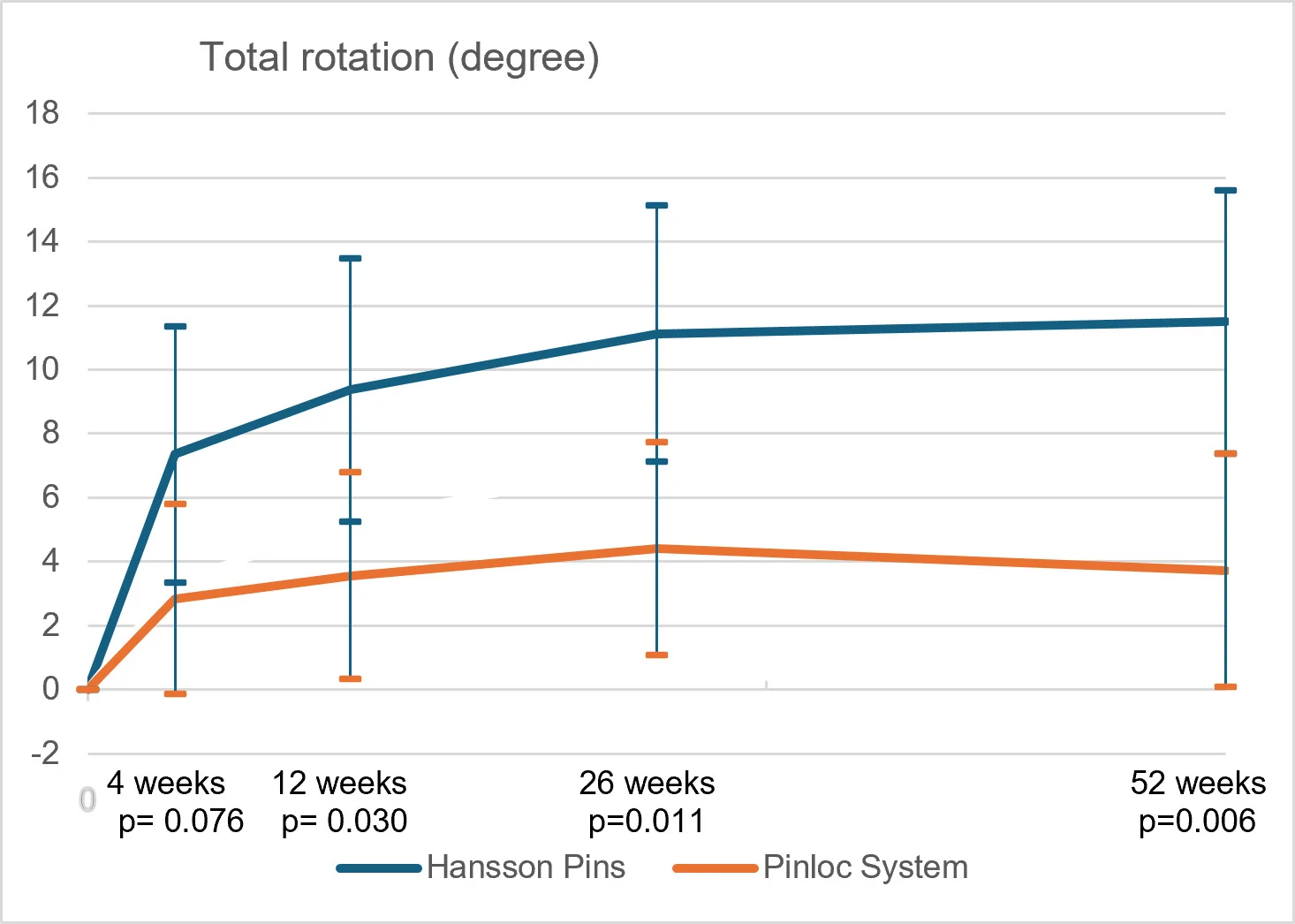
Graph showing mean total rotation for both groups, with 95% CIs.

Total rotational displacement at 52 weeks was lower in the Pinloc group than in the pin group (mean difference (MD) −7.77° (95% CI −13.27 to −2.28); p = 0.006) (Table III). The difference in total rotation was evident at 12 weeks and persisted at later follow-up (Figure 3). No significant differences were observed for rotation about any individual axis at any timepoint (Supplementary Material).

**Table III.** Migration analysis from after weightbearing to 52 weeks postoperatively (mean 385 days (SD 50)).

Variable	Pin group (n = 5)	Pinloc group (n = 4)	MD (95% CI)	p-value*
**Mean fracture translation, mm (95% CI)**				
X-axis (+ medial/- lateral)	−0.84 (−1.80 to 0.12)	−0.64 (−1.42 to 0.14)	0.19 (−1.04 to 1.43)	0.758
Y-axis (+ proximal/- distal)	−4.11 (−6.59 to −1.63)	−3.45 (−5.42 to −1.49)	0.66 (−2.51 to 3.82)	0.685
Z-axis (- anterior/+ posterior)	0.02 (−1.17 to 1.21)	−0.69 (−1.65 to 0.28)	−0.70 (−2.24 to 0.83)	0.368
Total translation	4.87 (2.47 to 7.27)	4.04 (2.15 to 5.93)	−0.83 (−3.89 to 2.22)	0.593
**Mean fracture rotation, ° (95% CI)**				
X-axis (- anterior/+ posterior)	5.51 (0.50 to 10.52)	0.20 (−3.87 to 4.27)	−5.31 (−11.76 to 1.15)	0.107
Y-axis (- anteversion/+ retroversion)	0.65 (−1.47 to 2.77)	0.35 (−1.69 to 2.39)	−0.30 (−3.24 to 2.64)	0.841
Z-axis (- varus/+ valgus)	−3.42 (−6.81 to −0.04)	−0.64 (−3.58 to 2.31)	2.79 (−1.70 to 7.28)	0.223
Total rotation	11.50 (7.39 to 15.61)	3.72 (0.08 to 7.38)	−7.77 (−13.27 to −2.28)	0.006

Marker configuration and accuracy parameters for the RSA segments,presented as mean (SD) in both caput and trochanter segments: Markers: caput 7.8 (1.8), trochanter 9.1 (1.4). Condition number: caput 79.0 (22.4), trochanter 42.8 (12.0). Mean error of rigid-body fitting (ME): caput 0.22 (0.09), trochanter 0.21 (0.06).

* Linear mixed model.

MD, mean difference.

**Fig. 4:**
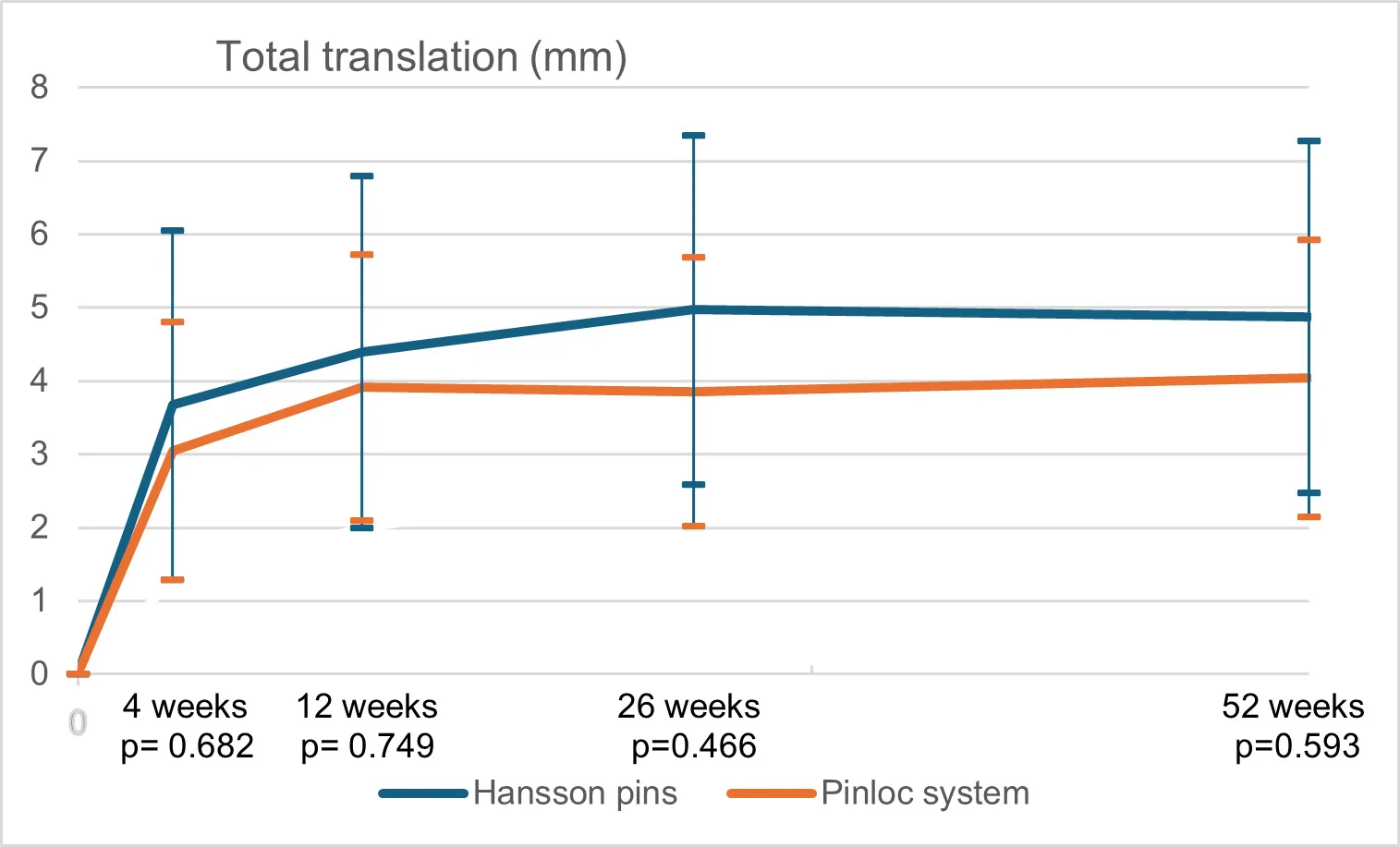
Graph showing mean total translation for both groups, with 95% CIs.

Total translation at 52 weeks was lower in the Pinloc group, but the between-group difference was not statistically significant (MD −0.83 mm (95% CI −3.89 to 2.22); p = 0.593) (Table III). No differences in total translation were observed at any other follow-up timepoint (Figure 4), and no axis-specific translations differed between groups (Supplementary Material).

In terms of correlation with bone density, linear mixed-model analysis showed no statistically significant association between the DXA T-score and total rotation or total translation. The estimated effect for total rotation was -1.87° per unit change in T-score (95% CI -4.87 to 1.12, p = 0.194). For total translation the estimated effect was -0.19 mm per unit change in T-score (95% CI -1.83 to 1.45, p = 0.803).

The median operating time was longer in the Pinloc group than in the pin group (51 mins (IQR 45 to 53) vs 35 mins (IQR 27 to 43), p = 0.015, Mann-Whitney U test).

Three patients died during follow-up from causes unrelated to the hip fracture (two in the Pinloc group and one in the pin group) (Figure 2). All patients, except for one in the pin group, demonstrated mechanical stabilization by RSA within 26 weeks. In all cases, fractures that demonstrated mechanical union also met the criteria for clinicoradiological healing. For the one patient in the pin group, the clinicoradiological criteria for union were met at the 52 weeks follow-up.

A total of five patients required reoperation: two in the pin group due to avascular necrosis, three in the Pinloc group due to avascular necrosis (n = 1), and sub-trochanteric fracture after low-energy trauma (n = 2).

PROMs showed no significant differences between groups at 52 weeks (Table IV).

**Table IV.** Median clinical outcomes and IQRs 52 weeks after surgery.

52-week outcomes	Pin group (n = 9)	Pinloc group (n = 7)	p-value*
Harris Hip Score	79 (75 to 93)	71 (63 to 88)	0.640
EQ-5D VAS	80 (70 to 90)	75 (50 to 80)	0.555
EQ-5D Index	0.76 (0.71 to 1)	0.71 (0.66 to 1)	0.299
VAS Pain	0 (0 to 1)	2 (1 to 4.5)	0.730
VAS satisfaction	9 (8 to 9.5)	8 (7 to 8)	0.795
TUG test, sec	17 (6 to 28)	10 (7 to 15)	0.120

* Linear mixed model.

EQ-5D, EuroQol five-dimension questionnaire; TUG, timed up and go; VAS, visual analogue scale.

Exploratory analyses of pre- to post-mobilization RSA showed minimal but measurable fracture motion (Table V).

**Table V.** Fracture motion pre- to post-mobilization (Pinloc group = 6, pin group = 1).

Variable	Pin group (n = 1) + Pinloc group (n = 6), mean (95% CI)	Pin group (n = 1), mean	Pinloc group (n = 6), mean
**Fracture translation, mm**			
X-axis	−0.16 (−0.38 to 0.07)	−0.04	−0.18
Y-axis	−0.28 (−0.49 to −0.06)	−0.23	−0.28
Z-axis	−0.01 (−0.53 to 0.51)	0.10	−0.03
Total translation	0.58 (0.21 to 0.94)	0.26	0.63
**Fracture rotation, °**			
X-axis	−0.02 (−0.45 to 0.42)	0.02	−0.02
Y-axis	0.19 (−1.11 to 1.49)	−0.78	0.35
Z-axis	−0.13 (−0.38 to 0.12)	−0.32	−0.10
Total rotation	1.20 (0.44 to 1.96)	0.84	1.26

## Discussion

Hansson Pinloc plate fixation was associated with increased torsional stability compared with Hansson pin fixation, with a statistically significant reduction in total rotational displacement evident from 12 weeks. Both groups demonstrated translational movement with shortening and reduced offset.

The relevance of torsional stability in the internal fixation of femoral neck fractures has been debated for decades.^23,24^ While stability is biomechanically necessary for uneventful healing according to the strain theory,^25^ the clinical relevance of increased torsional stability remains uncertain. A recent study of distal femur fractures demonstrated that improved construct stability does not necessarily translate into better clinical outcomes.^26^ Although our data were under-powered for evaluating clinical outcomes, we observed no clear benefit of increased torsional stability on fracture healing or functional recovery. This is consistent with a larger randomized controlled trial (RCT) comparing Hansson Pins with the Pinloc system showing no clinically relevant differences at one- and two-year follow-up.^13,27^

Given the precision of RSA, the observed difference in rotation is unlikely to be fully explained by measurement error alone. The baseline differences were small, and between-group differences were limited, suggesting a potential true difference in rotational stability, whereas the clinical impact remains uncertain.

Conventional implants generally permit fracture compression as a mechanism for fracture union. We observed a similar pattern of translational behaviour in both groups with reduced mean leg length and reduced offset. It has been suggested that some rotational instability may be necessary for adequate compression;^4^ our results indicate that increased stability did not appear to compromise this process. Fracture motion was greatest during the first four weeks and stabilized by 12 weeks in almost all cases (Figure 2 and Figure 3), with shortening in translation and transverse-axis rotation being most prominent, consistent with previous findings.^28^ For the small sub-group with premobilization images, only minimal movement occurred before weightbearing, in line with a previous report.^29^

Concerning fracture healing, mechanical healing was observed in all but one fracture at 26 weeks, consistent with previous RSA results.^28^ However, this study was not designed to detect differences in healing time between the groups.

While locking plates that restrict fracture compression are associated with high complication rates,^9^ this mechanism is less likely with interlocked pins, as translational fracture compression is maintained. However, sub-trochanteric fractures occurred in the Pinloc group, a complication also reported previously.^30^ Connecting three pins though a lateral plate may increase the stress on the sub-trochanteric region potentially contributing to higher incidence of sub-trochanteric fractures,^13^ consistent with an ex-vivo report of facilitated load transfer from medial to lateral with the Pinloc system.^31^ However, the small sample size precludes firm conclusions regarding the clinical relevance of these observations. In line with previous reports suggesting an increased risk of sub-trochanteric fractures,^30^ the manufacturer has adjusted the plate angle from 125° to 120° to elevate the entry points of the plate and screws.

Although the Hansson Pin group had a shorter median operating time, the difference is unlikely to be clinically relevant. The duration in this group likely reflects the pursuit of optimal implant positioning. Additionally, the observed AVN rate of 12% is slightly higher than expected, which may reflect small sample size and close follow-up. Similarly, the lack of correlation between BMD and migration may be because the study was under-powered to detect such associations.

To our knowledge, this is the first in-vivo RSA study reporting increased torsional stability and reproduces the reported failure pattern previously demonstrated in ex-vivo investigations.^11,12^ These findings bridge biomechanical models and clinical observations; our findings suggest that the interlocking principle reduced rotational movement. While small sample size warrants caution, this mechanical rationale complements the existing clinical RCT by suggesting that increased torsional stability may not be associated with improved functional recovery in stable femoral neck fractures.^13,27^

A substantial number of RSA examinations were excluded for not meeting predefined RSA quality criteria (ME < 0.35 and CN < 150), likely due to suboptimal marker distribution and marker instability in osteoporotic bone. This reduced the sample below the planned power, increasing the risk of type I and II statistical errors, and results should be interpreted with caution. The RSA precision was comparable with a previous RSA study of hip fractures.^22^ To assess potential selection bias, patients with and without 52-week RSA data were compared. No significant differences were found in baseline demographic details or PROMs (four weeks), indicating no clear evidence of systematic differences between groups with complete and incomplete follow-up. Furthermore, the use of a LMM may have mitigated some of the loss of power by utilizing all longitudinal data. However, the absence of a formal screening log limits the ability to fully assess the inclusion process and may introduce a risk of selection bias. The study was under-powered for clinical outcomes. Fracture classification used plan radiographs only, and posterior comminution that could have been detected by CT may have been missed.

Furthermore, the study population consisted of older patients, a group increasingly treated with arthroplasty rather than internal fixation.^2^ This limits the clinical relevance of our findings, as younger or more physiologically fit patients may represent a more suitable cohort for evaluating the potential advantages of improved fixation stability.

In conclusion, our findings show that the Hansson Pinloc system provided increased torsional stability compared with conventional pins during healing of undisplaced femoral neck fractures, confirming a mechanical effect on in-vivo fracture behaviour. However, this increased stability was not associated with differences in fracture healing or clinical outcomes in older patients with undisplaced femoral neck fractures. In perspective, these findings suggest that greater construct stiffness alone may not be sufficient to improve outcomes in this fracture type.

**Take home message**

- Interlocking plate fixation (Hansson Pinloc) provided increased torsional stability compared with conventional pin fixation in undisplaced femoral neck fractures, measured by radiostereometric analysis.

- No difference in clinical outcomes or reoperation was detected, although the study was under-powered and findings should be interpreted with caution.

- The clinical relevance of increased torsional stability in undisplaced fractures remains uncertain.

## Data Availability

The datasets generated and analyzed in the current study are not publicly available due to data protection regulations. Access to data is limited to the researchers who have obtained permission for data processing. Further inquiries can be made to the corresponding author.
